# IPSE, a urogenital parasite-derived immunomodulatory molecule, suppresses bladder pathogenesis and anti-microbial peptide gene expression in bacterial urinary tract infection

**DOI:** 10.1186/s13071-020-04490-8

**Published:** 2020-12-09

**Authors:** Evaristus C. Mbanefo, Loc Le, Luke F. Pennington, Yi- Ju Hsieh, Justin I. Odegaard, Kristina Lapira, Theodore S. Jardetzky, Franco H. Falcone, Michael H. Hsieh

**Affiliations:** 1 Division of Urology, Department of Surgery, Children’s National Hospital, West Wing, 4th Floor, 111 Michigan Avenue NW, Washington, DC 20010 USA; 2 Biomedical Research Institute, Rockville, MD USA; 3 Department of Structural Biology, Stanford University, Stanford, CA USA; 4 Guardant Health, Redwood City, CA USA; 5 University of California San Diego, La Jolla, USA; 6 Institute of Parasitology, Justus-Liebig-Universität Gießen, Gießen, Germany; 7 Present Address: National Institutes of Health, Bethesda, MD USA; 8 Present Address: A-TEK, Baltimore, MD USA; 9 Present Address: Fountain Biopharma, Taipei, Taiwan

**Keywords:** Schistosome, Schistosoma, Haematobium, IPSE, α-1, Bladder, Urinary tract infection, IL-4

## Abstract

**Background:**

Parasitic infections can increase susceptibility to bacterial co-infections. This may be true for urogenital schistosomiasis and bacterial urinary tract co-infections (UTI). We previously reported that this co-infection is facilitated by *S. haematobium* eggs triggering interleukin-4 (IL-4) production and sought to dissect the underlying mechanisms. The interleukin-4-inducing principle from *Schistosoma mansoni* eggs (IPSE) is one of the most abundant schistosome egg-secreted proteins and binds to IgE on the surface of basophils and mast cells to trigger IL-4 release. IPSE can also translocate into host nuclei using a nuclear localization sequence (NLS) to modulate host transcription. We hypothesized that IPSE is the factor responsible for the ability of *S. haematobium* eggs to worsen UTI pathogenesis.

**Methods:**

Mice were intravenously administered a single 25 μg dose of recombinant *S. haematobium-*derived IPSE, an NLS mutant of IPSE or PBS. Following IPSE exposure, mice were serially weighed and organs analyzed by histology to assess for toxicity. Twenty-four hours after IPSE administration, mice were challenged with the uropathogenic *E. coli* strain UTI89 by urethral catheterization. Bacterial CFU were measured using urine. Bladders were examined histologically for UTI-triggered pathogenesis and by PCR for antimicrobial peptide and pattern recognition receptor expression.

**Results:**

Unexpectedly, IPSE administration did not result in significant differences in urine bacterial CFU. However, IPSE administration did lead to a significant reduction in UTI-induced bladder pathogenesis and the expression of anti-microbial peptides in the bladder. Despite the profound effect of IPSE on UTI-triggered bladder pathogenesis and anti-microbial peptide production, mice did not demonstrate systemic ill effects from IPSE exposure.

**Conclusions:**

Our data show that IPSE may play a major role in *S. haematobium*-associated urinary tract co-infection, albeit in an unexpected fashion. These findings also indicate that IPSE either works in concert with other IL-4-inducing factors to increase susceptibility of *S. haematobium-*infected hosts to bacterial co-infection or does not contribute to enhancing vulnerability to this co-infection.
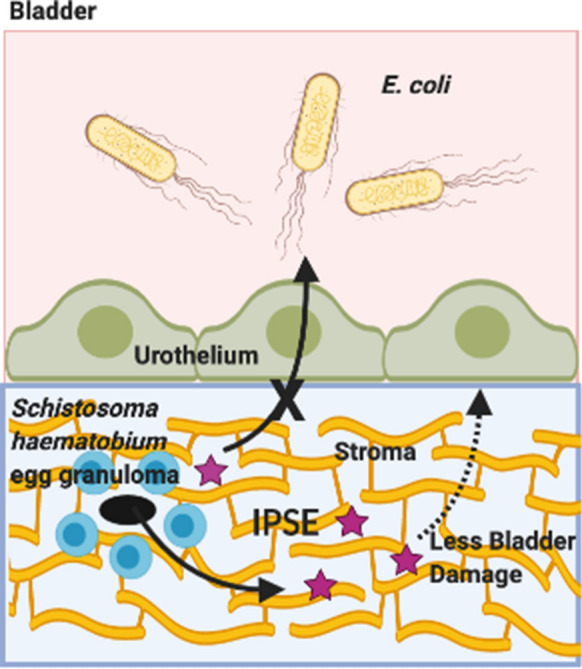

## Background

Helminths have co-evolved with humans to immunomodulate their hosts. This includes schistosomes, which affect at least 200 million people in Brazil, sub-Saharan Africa, the Middle East, and Asia [1]. The main schistosomes affecting humans include *Schistosoma mansoni*, *Schistosoma haematobium*, and *Schistosoma japonicum*, with *S. haematobium* comprising the majority of infections. All three of these species secrete immunomodulatory proteins, such as omega-1 [2], kappa-5 [3], SJHME-1 [4], Sj16 [5], and the interleukin-4-inducing principle of *Schistosoma mansoni* eggs (IPSE) [6], also called α-1 [7]. These immunomodulatory products of schistosomes have been postulated to exert far-reaching regulatory effects on host responses not only to schistosomiasis, but also co-infections. Schistosomiasis is co-endemic with many other pathogens, including *Plasmodium* [8], HIV [8], mycobacteria [9], other helminths and parasites [10], and also *Enterobacteriaceae* such as *Salmonella* [11]. Another member of *Enterobacteriaceae* that may co-infect large numbers of patients with schistosomiasis is *E. coli*.

Of the various types of disease-causing *E. coli*, uropathogenic strains are a common cause of infection in males and females, ranging from neonates to the elderly. One-quarter of all girls and women will experience multiple bacterial urinary tract infections (UTI) [12], and up to half of all girls and women will suffer from a bacterial UTI at least once in their lifetimes [13]. Several reports have observed that patients with urogenital schistosomiasis are more prone to bacterial UTI [14–16], although two papers have disputed this association [17, 18]. The potential causal link between urogenital schistosomiasis and UTI has been debated. One hypothesis is that *S. haematobium* infection leads to immune polarization, which is detrimental to clearance of bacteriuria. Indeed, we have demonstrated in a mouse model that *S. haematobium* eggs injected into the bladder wall (a primary tissue site containing eggs in patients with urogenital schistosomiasis) increase susceptibility to subsequent UTI [19]. This increased susceptibility is associated with egg-induced, IL-4-driven suppression of natural killer T (NKT) cell activity. The role of NKT cells was confirmed by restoring the activity of this cellular subset using α-galactosylceramide, an NKT cell agonist. Thus, the heightened vulnerability of patients with urogenital schistosomiasis to UTI may be immunologically mediated.

The egg-associated factors which orchestrate this IL-4-dependent phenomenon may include the interleukin-4-inducing principle of *Schistosoma mansoni* eggs (IPSE) [6], also known as α-1 [7]. IPSE is one of the most abundant schistosome egg-secreted proteins [20] and features multiple immunomodulatory properties. First, IPSE binds to Fcε receptor-bound IgE on the surface of basophils and mast cells to trigger IL-4 secretion [21–23]. It is also able to ligate immunoglobulins on the surface of B regulatory cells (Bregs) and subsequently activate these cells [24]. The *S. mansoni* ortholog of IPSE named *S. mansoni* chemokine-binding protein (smCKBP) can neutralize chemokines [25]. Lastly, IPSE features a nuclear localization sequence which guides the protein to host cell nuclei [26, 27], where it modulates transcription [28, 29].

We hypothesized that IPSE may be the key IL-4-inducing principle of *S. haematobium* eggs responsible for increased UTI susceptibility in affected hosts. Our interest in testing this hypothesis was based on both a wish to better understand the pathogenesis of urogenital schistosomiasis and a desire to learn the safety profile of IPSE as a potential therapeutic. In past work we have demonstrated that IPSE is a potent anti-inflammatory agent for ifosfamide-induced hemorrhagic cystitis [21, 29, 30].

Herein, we report that although IPSE did not affect levels of bacteriuria in UTI-challenged mice, IPSE exposure did lead to markedly reduced UTI-triggered bladder pathogenesis and a decrease in expression of anti-microbial peptides. Finally, IPSE was not associated with any discernable adverse systemic effects, thus leaving the path open for further development of this molecule as a potential therapeutic.

## Methods

### Study approval

All animal work was conducted according to relevant US and international guidelines. Specifically, animal experimental work was reviewed and approved as protocol 14-03 by the Institutional Animal Care and Use Committee of the Biomedical Research Institute (Rockville, MD, USA). Our Institutional Animal Care and Use Committee guidelines comply with the US Public Health Service Policy on Humane Care and Use of Laboratory Animals.

### Mice

Six to 7-week-old male and female BALB/c mice (Charles River Laboratories, Wilmington, MA, USA) were housed under 12 h light-dark cycles in temperature-controlled holding rooms with unlimited access to dry mouse chow and water. Newly received mice were acclimated to the animal facility for at least 1 week prior to experimental use.

### IPSE protein production

Recombinant H06 H-IPSE (one of the major *Schistosoma haematobium* IPSE orthologs) and an NLS mutant of H06 H-IPSE (the wild-type NLS sequence SKRGRKY changed to SAAGAAY) were produced in HEK293-6E cells as previously described [27].

### IPSE administration

One day prior to UTI induction, mice underwent tail vein injection with phosphate-buffered saline or 25 μg of H06 H-IPSE (or its NLS mutant) in phosphate-buffered saline.

### UTI89 infection of mice

The uropathogenic *Escherichia coli* (UPEC) strain UTI89 was derived from a patient with cystitis [31]. Briefly, UTI89 was streaked on an LB agar plate to obtain a single colony which was then inoculated into 10 ml LB broth and grown at 37 °C overnight under static conditions to promote type 1-pilus expression [32, 33]. Bacteria were then subcultured 1:100 into 10 ml fresh medium followed by growth at 37 °C for another 18 h. These cultures were centrifuged for 10 min at 14,000 rpm, resuspended in 2 ml PBS, and then diluted to approximately 2 × 10^9^ CFU per ml as estimated using a spectrophotometer (OD_600 nm_); 50 μl of this suspension (10^8^ CFU) was inoculated into the bladders of female mice by transurethral catheterization [34]. Male mice were not tested for UTI susceptibility; catheterization of male mice is technically very challenging.

### Bacterial titer determinations

Freshly collected urine was serially diluted 100- and 1000-fold in sterile PBS. Twenty-five microliters of each dilution were plated onto MacConkey agar plates. CFU were counted after overnight incubation at 37 °C. The limits of detection were 4000 CFU/ml for urine.

### Histology

Bladder tissues were fixed in neutral buffered formalin, dehydrated, and embedded in paraffin. Five-micrometer sections were stained with hematoxylin and eosin. Histology was analyzed for each bladder by a board-certified pathologist (JIO) in a blinded fashion, using the following scoring system:

- Urothelium

0: Normal
1: Urothelial hyperplasia with reactive nuclear atypia and architectural disarray
2: Segmental urothelial ulceration

- Edema

0: Normal
1: Perivascular edema with expansion but preservation of bladder architecture
2: Severe edema with effacement of the submucosa

- Inflammation

0: Normal
1: Perivascular acute inflammatory infiltrates
2: Frank acute inflammation of the urothelium with urothelial microabscesses

- Hemorrhage

0: None
1: Sparse hemorrhage
2: Frank intra-tissue hemorrhage

- Contraction

0: Normal papillae
1: Blunted/wide contraction papillae
2: Effaced contraction papillae
  0.5 gradations for focality

### RNA purification

RNA was isolated from mouse bladders using TRIzol Reagent and PureLink RNA Mini Kit (Invitrogen), according to the manufacturers’ instructions. Briefly, aseptically excised bladders were homogenized in 1 ml TRIzol reagent by bead-beating using ceramic beads (Omni International) and a mini-bead beater (Biospec). Following a 5-min incubation, 0.2 ml chloroform was added and again incubated for 3 min before centrifugation at 12,000×*g* for 15 min to separate homogenates into aqueous and organic phases. The aqueous supernatant (~ 400 μl) was mixed with an equal volume of 70% ethanol before binding the mixture to RNA binding columns by centrifugation. On-column DNase digestion (Invitrogen) was performed for 30 min, following the manufacturer’s protocols. After column washes and drying, RNA was eluted in RNase-free water, quantified, and its quality checked using a NanoDrop 1000 spectrophotometer (Thermo Scientific) and Bioanalyzer 2100 (Agilent).

### cDNA synthesis and real-time PCR

cDNA synthesis was performed using the RT2 First Strand cDNA kit (SABiosciences). Real-time PCR was performed for anti-microbial peptide gene expression using an Mx3005p thermocycler (Stratagene) using an RT2 custom PCR array (SABiosciences) with RT2 SYBR green quantitative PCR (qPCR) master mixes (SABiosciences). Cycle thresholds (*C*_T_) were calculated for each reaction. Using the comparative *C*_T_ method, relative gene expression was calculated as $2^{{\left( { - \Delta \Delta C_{{\text{T}}} } \right)}}$, where Δ*C*_T_ = *C*_T_ (gene of interest) − Δ*C*_T_ (β-actin). ΔΔ*C*_T_ was calculated as Δ*C*_T_ (H-IPSE injection and bacterially infected) − Δ*C*_T_ (bacterially infected). Data are expressed as means ± standard deviations (SDs). *P* values were calculated using Mann-Whitney *U* tests comparing Δ*C*_T_ of the groups receiving H-IPSE and UTI challenge to the groups receiving UTI challenge alone (*p < 0.05; **p < 0.01; ***p < 0.001). Melt curves confirmed the specificity of PCR reactions. PCR primer sequences are shown in Additional file 1: Table S1.

### Toxicity assays

Toxicity assays were performed using four groups including one vehicle control (phosphate-buffered saline) and three H-IPSE treated groups (0.5, 1, and 2 mg/kg/day via tail vein injection daily for 7 days), using BALB/c mice. The dosing volume was kept constant at 6.35 ml/kg/day for each mouse. Parameters evaluated included clinical signs of illness, body weight, percent body weight gain, feed consumption, hematology, clinical chemistry, and, after 7 days, organ weights, gross pathology, and histopathology.

### Histamine measurements

Two groups of mice (BALB/c 6-week old, *n* = 4 in each group) were injected with either PBS or 25 μg H-IPSE intravenously. Mice were bled 5 min after injection with PBS or H-IPSE via cheek bleed and 30 min post injection via terminal heart bleed. Sera were collected and immediately used for histamine analysis using a mouse histamine ELISA kit from MyBioSource using the manufacturer’s instructions (catalog no. MBS725193).

### Statistical analysis

Statistical analyses were performed using GraphPad software. Except where noted otherwise, Mann-Whitney *U* tests and Student *t*-tests were used to evaluate statistical significance for nonparametrically and parametrically distributed data, respectively. ANOVAs with Tukey’s multiple comparison test were used for comparisons of three or more experimental groups. *P* values < 0.05 were defined as significant.

## Results

### IPSE does not alter susceptibility to UTI

**Fig. 1:**
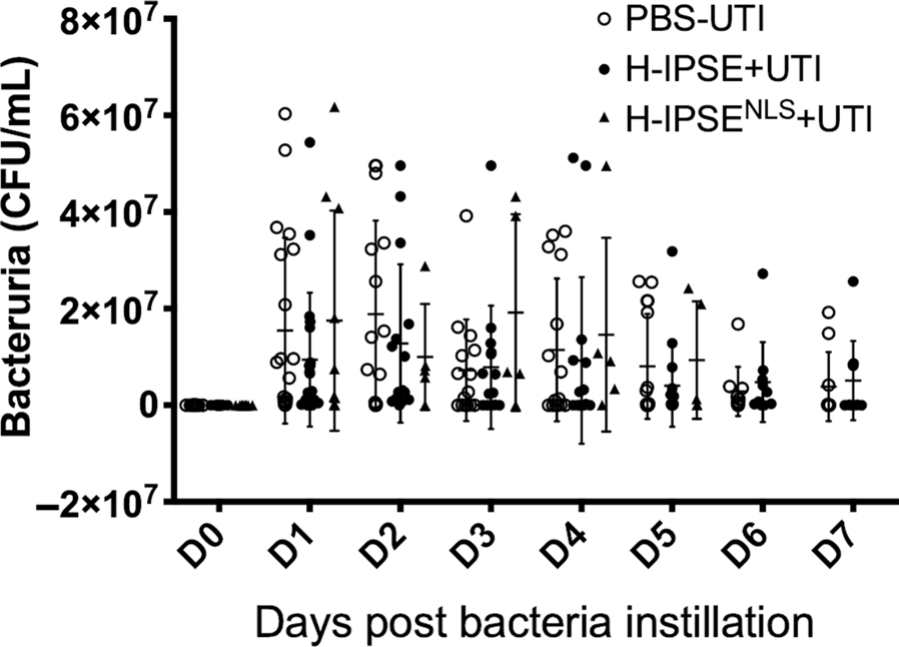
H-IPSE does not affect bacteriuria in mice. “PBS-UTI”: mice given PBS vehicle tail vein injections prior to UTI challenge; “H-IPSE+UTI”: mice administered the H06 ortholog of IPSE before UTI challenge; “H-IPSE^NLS^-+UTI”: mice given a nuclear localization sequence mutant of the H06 ortholog of IPSE prior to UTI challenge. Bars represent mean and standard deviation. Data are pooled from two independent experiments with each symbol representing an individual mouse’s urine CFU at the indicated point in time. Mice receiving H-IPSE^NLS^ were only sampled until day 5 post-infection, whereas all other mice were sampled until day 7. Observations were not determined to be statistically significant

In prior work we have demonstrated that a single injection of *S. haematobium* eggs into the bladder walls of otherwise UTI-resistant BALB/c mice renders them much more sensitive to UTI [19]. Thus, herein we sought to determine whether H-IPSE is sufficient to confer increased susceptibility to UTI. Surprisingly, a high intravenous dose of H-IPSE (25 μg, Fig. 1) did not enhance vulnerability of mice to transurethral infection with the uropathogenic UTI89 *E. coli* strain. This was true over an entire week of daily urine sampling, and a nuclear localization sequence (NLS)-deficient mutant of H-IPSE also did not alter UTI susceptibility in mice.

### IPSE reduces UTI-associated bladder pathology

**Fig. 2:**
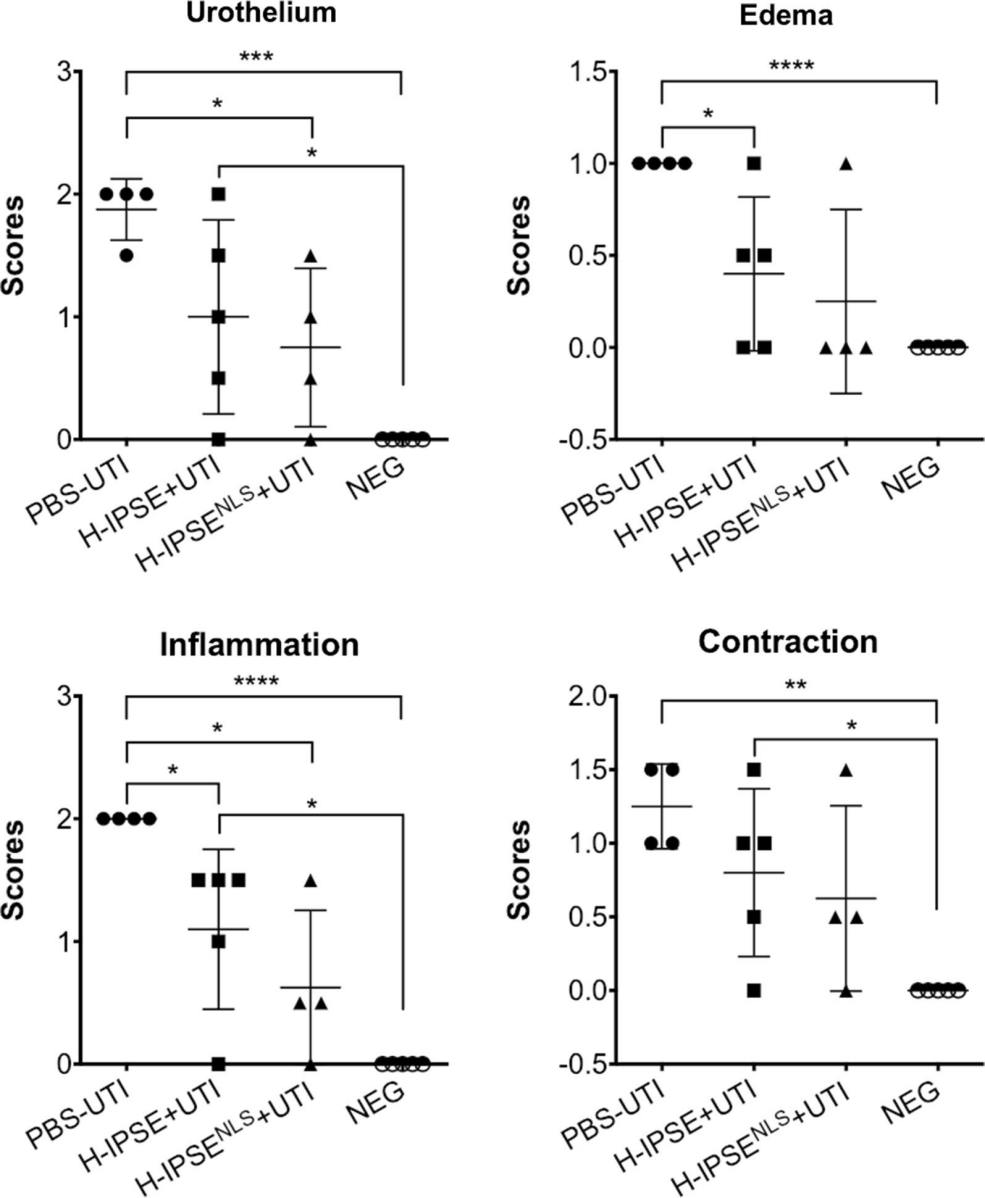
Effects of H-IPSE on UTI-associated bladder pathogenesis. H-IPSE reduces bladder pathogenesis. “PBS-UTI”: mice given PBS vehicle tail vein injections prior to UTI challenge; “H-IPSE+UTI”: mice administered the H06 ortholog of IPSE before UTI challenge; “H-IPSE^NLS^-+UTI”: mice given a nuclear localization sequence mutant of the H06 ortholog of IPSE prior to UTI challenge; “NEG”: unmanipulated mice. ANOVAs with Tukey’s multiple comparisons test were used for comparisons between group means with every other group. Bars represent means and standard deviation. Findings were considered statistically significant at *p* < 0.05. *, **, ***, and **** denote *p* < 0.05, *p* < 0.01, *p* < 0.001, and *p* < 0.0001, respectively. Data are representative of two independent experiments

In past work we have reported that H-IPSE dampens ifosfamide-triggered bladder pathogenesis. Hence, we tested whether H-IPSE alleviates UTI-induced bladder pathology. A single intravenous dose of H-IPSE indeed reduced bladder edema and inflammation, but not urothelial denudation or contraction (Fig. 2).

### IPSE reduces expression of anti-microbial peptides in the bladder

**Fig. 3:**
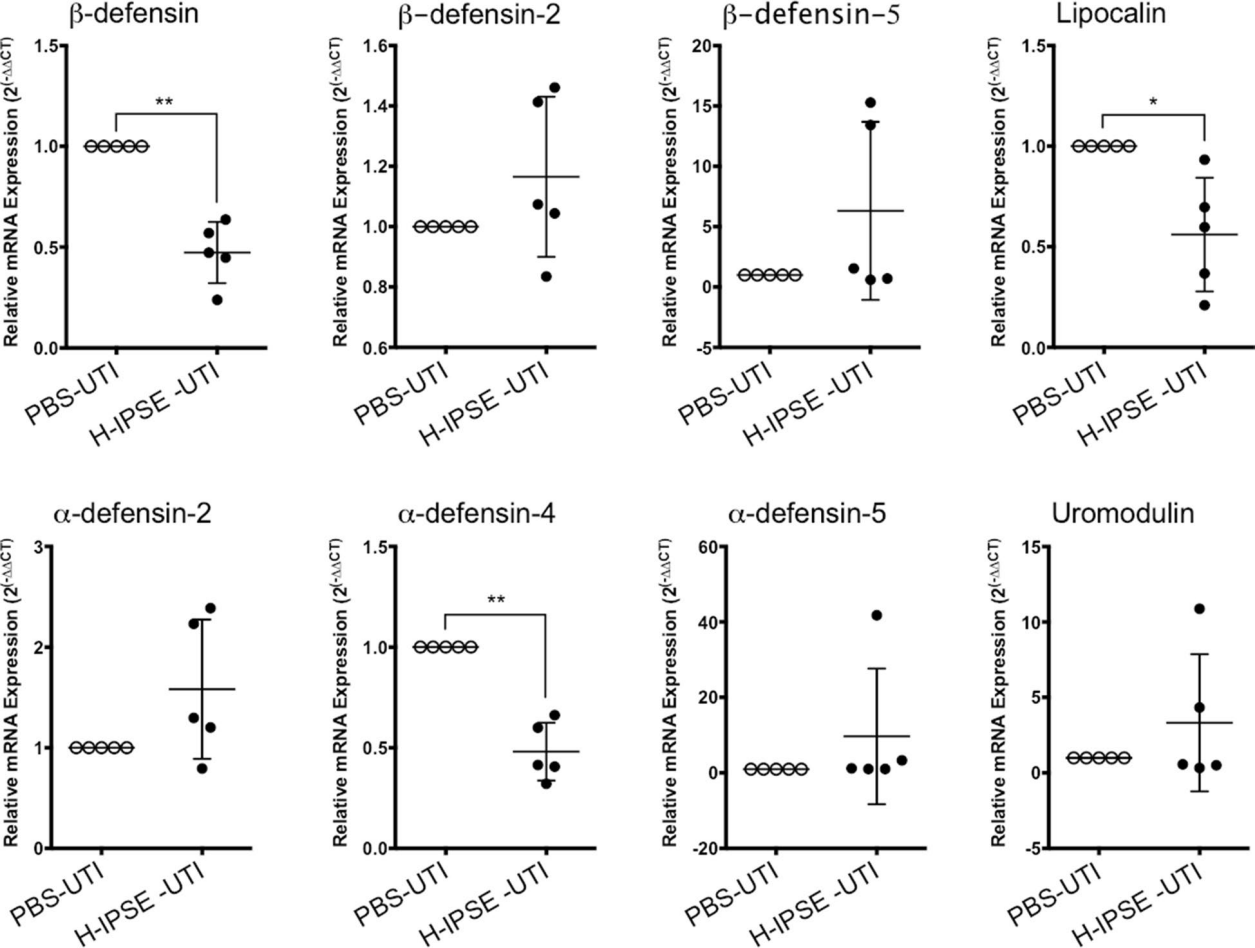
H-IPSE administration prior to UTI challenge decreases anti-microbial peptide gene expression in the mouse bladder. “PBS-UTI”: mice given PBS vehicle tail vein injections prior to UTI challenge; “H-IPSE+UTI”: mice administered the H06 ortholog of IPSE before UTI challenge. PCR on bladder RNA was performed 7 days post UTI challenge. Error bars represent mean and standard deviation. Differences between groups were considered statistically significant at *p* < 0.05. * and ** denote *p* < 0.05 and *p* < 0.01, respectively. Data are representative of two independent experiments

Considering that H-IPSE ameliorated UTI-triggered bladder inflammation, we postulated that this molecule may also modulate bladder production of anti-microbial peptides, which are part of the inflammatory response to infections. Amongst anti-microbial peptides assayed, three of them featured reduced gene expression following H-IPSE exposure (lipocalin, beta-defensin, and alpha-defensin-4, Fig. 3). TLR5, TLR4, LL-37, IL17a, IL12b, IL12a, IL10, IL4, IFNa, G-CSF, eNOS3, c-type lectin, and a-defensin 1 were also assayed by qPCR but did not show statistically significant trends (data not shown).

### IPSE does not increase circulating histamine

**Fig. 4:**
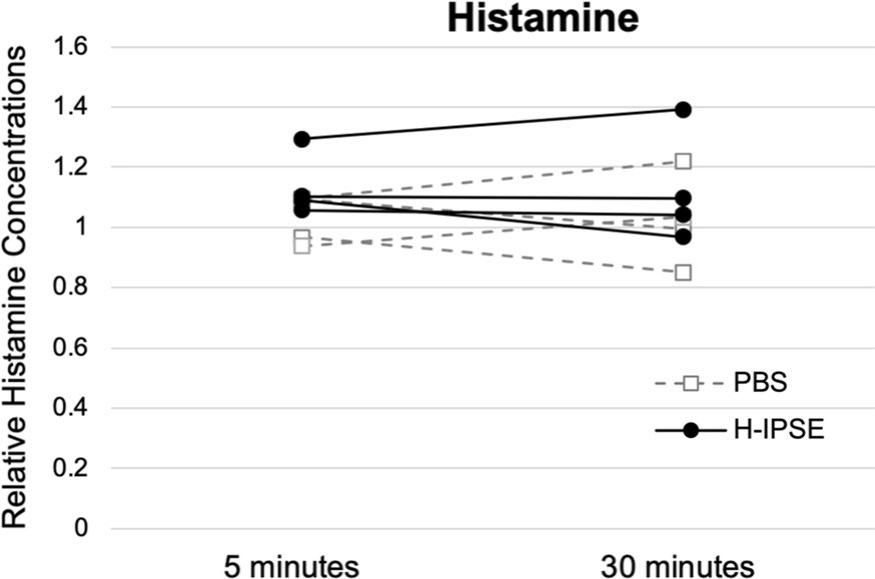
Intravenously administered H-IPSE does not increase circulating histamine levels. “PBS”: mice injected with phosphate buffered saline; “H-IPSE”: mice injected with H-IPSE. Circles and solid black lines represent individual mice that received H-IPSE injection while squares and gray dashed lines represent PBS-injected mice. No significant differences were found in the levels of circulating histamine between PBS- or H-IPSE-injected groups at either 5 min (*p* = 0.15) or 30 min (*p* = 0.44). Data representative of two independent experiments

IPSE is known to ligate Fcε receptor-bound IgE on the surface of basophils and mast cells [21–23], which could conceivably lead to histamine release and anaphylaxis. We therefore tested the influence of intravenous H-IPSE on circulating histamine in mice. These assays revealed that systemic histamine levels are not increased in H-IPSE-exposed mice at 5 or 30 min post-IPSE injection (Fig. 4).

### IPSE does not cause systemic pathology

**Fig. 5:**
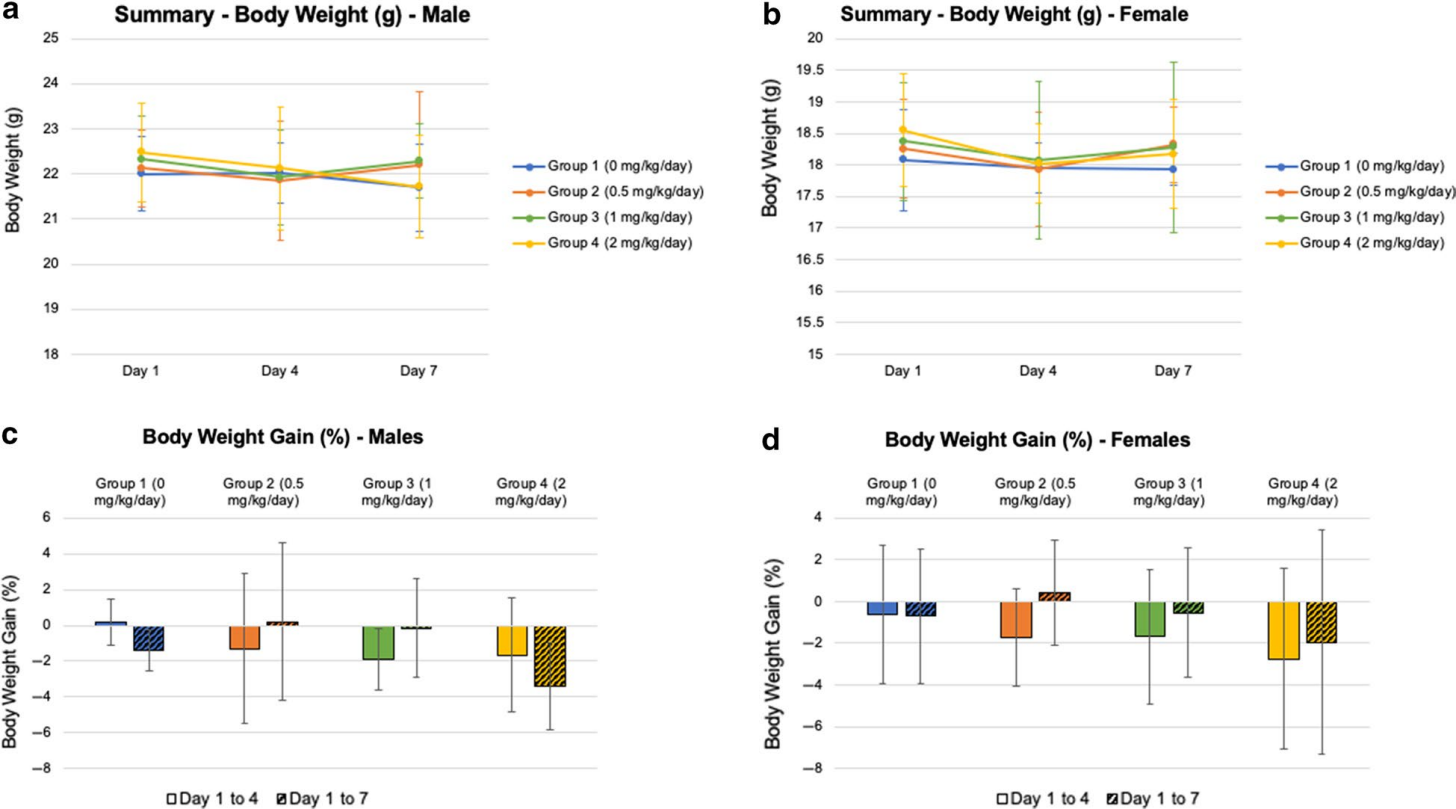
Treatment with H-IPSE at doses up to 2 mg/kg/day had no effect on body weight and body weight gain. Mice were injected with varying concentrations of H-IPSE, and the body weights of males (**a**) and females (**b**) were monitored over 7 days. Changes in percent body weight gains for male (**c**) and female (**d**) mice were not significant. Solid-colored bars represent average body weight gain (%) during the first 4 days while patterned colored bars represent total change over the course of the experiment (7 days). Error bars represent standard deviation and *n* = 3 for each group, with results showing pooled results of two separate replicate experiments for a total of *n* = 6 for each group

Taking into account IPSE’s multi-faceted immunomodulatory effects on the host, our next objective was to determine if H-IPSE exposure leads to systemic pathology. Intravenous administration of H-IPSE at doses of 0.5, 1, and 2 mg/kg/day to mice did not lead to any abnormal clinical signs in mice. All animals survived to study termination. Treatment with H-IPSE at doses up to 2 mg/kg/day had no effect on body weight and body weight gain (Fig. 5), feed consumption, hematology, clinical chemistry, or gross pathology (see Additional file 1: Tables S2–S21). Clinical chemistry analytes of all H-IPSE-treated animals were comparable to control animals, except increased globulin (by 1.1-fold) and decreased albumin globulin (A:G) ratio (by 1.2-fold) at 0.5 mg/kg/day in females. Organ weights of all H-IPSE-treated animals were comparable to control animals, except increased relative weights of liver at 1 mg/kg/day in females. Histopathological evaluation of mice treated with H-IPSE did not show any lesions of pathological significance except for minimal focal basophilic tubules in kidneys in one male and one female mouse receiving either control vehicle or 2 mg/kg/day of H-IPSE (i.e. four mice total), minimal focal neutrophil and lymphocytes infiltration at the site of injection (one male mouse receiving vehicle and one female mouse receiving 2 mg/kg/day of H-IPSE), minimal focal extramedullary hematopoiesis in the liver of one vehicle-treated female mouse, minimal focal cystic spaces in the liver of one female mouse receiving 2 mg/kg/day of H-IPSE, and minimal unilateral atrophy of the uterus in two female mice receiving 2 mg/kg/day of H-IPSE.

## Discussion

Whether a causal connection between urogenital schistosomiasis and bacterial UTI exists [14–16, 35, 36] or not [17, 18, 37] has been debated by several groups. Multiple hypotheses could explain a potential cause-and-effect relationship between *S. haematobium* infection, the primary cause of urogenital schistosomiasis, and UTI. It is possible, for instance, that detection bias contributes to increased reporting of UTI among patients with urogenital schistosomiasis. In this scenario, increased urine testing and urinary symptom tracking of individuals living in endemic areas lead to incidental discovery of UTI. Another potential hypothesis explaining a causal link between urogenital schistosomiasis and UTI is related to urinary tract obstruction. Schistosome egg deposition in the bladder initiates granulomatous fibrosis. In severe cases, this fibrosis can obstruct the urinary tract, contributing to urine stasis and bacterial overgrowth. Finally, it is possible that *S. haematobium* infection leads to immune skewing, which hinders host clearance of bacteriuria. In support of this hypothesis, we showed that *S. haematobium* eggs injected into the mouse bladder wall renders BALB/c mice, which are typically UTI-resistant, highly sensitive to subsequent UTI [19]. The ability of *S. haematobium* eggs to enhance susceptibility to urinary tract co-infection relies upon IL-4 and also depended upon this cytokine dampening NK T cell activity. These findings lend credence to the possibility that the increased sensitivity of patients with urogenital schistosomiasis to UTI is orchestrated by schistosome-based immune modulation.

Based on IPSE’s capacity to trigger IL-4 secretion and modulate host immunity more broadly, we posited that mouse exposure to *S. haematobium-*derived H-IPSE would be sufficient to augment UTI sensitivity. We sought to examine this postulate for two reasons. First, we wished to delineate H-IPSE’s immune mechanisms given its association with urogenital schistosomiasis. We also sought to ascertain whether and how H-IPSE would be efficacious against detrimental, UTI-associated bladder inflammation. This goal was based on our past observations that a single injection of H-IPSE prevents severe bladder inflammation triggered by ifosfamide exposure [21, 29, 30].

Taking into account H-IPSE’s profound effects on other types of bladder inflammation, we were surprised by our finding that H-IPSE did not alter clearance of subsequent UTI. Specifically, H-IPSE did not change mean bacterial CFU over a week of daily urine sampling. H-IPSE also did not modify the distribution of bacterial CFU; in the mouse UTI model, sometimes mice in the same treatment group can cluster into bimodal distributions in terms of magnitude of bacteriuria. Monitoring levels of bacteriuria serially over multiple time points is important considering that the temporal kinetics of the mouse UTI model are highly dynamic. Our findings indicate that despite H-IPSE’s multi-faceted effects on host immunity, this molecule neither helps nor hinders clearance of bacteriuria.

Although H-IPSE did not affect bacteriuria, it did alter UTI-induced bladder pathology. Namely, H-IPSE decreased bladder inflammation and edema, but did not ameliorate bladder contraction or urothelial denudation. By comparison, we have previously shown that H06-IPSE, the same ortholog tested in this study, reduces ifosfamide-induced bladder hemorrhage and histologically evident edema, but not bladder wet weights [21]. In another publication we observed that H03-IPSE, another major *S. haematobium* ortholog, dampens ifosfamide-induced bladder wet weight increases [30]. We did not measure bladder hemorrhage and wet weights in this study because bacterial UTI does not significantly alter these parameters (data not shown). It is noteworthy that NLS-IPSE also reduced urothelial pathology and bladder inflammation. NLS-IPSE may be mediating these effects through H-IPSE’s chemokine sequestering and/or IL-4-inducing capabilities. Certainly, H-IPSE’s alleviation of UTI-triggered bladder inflammation is consistent with its known immunomodulatory properties.

After observing H-IPSE’s effects on UTI-associated bladder inflammation, we measured this molecule’s impact on bladder expression of anti-microbial peptides, an important set of immune responses to infection that evolved in early eukaryotes. Of the ten anti-microbial peptides examined, gene expression of lipocalin, beta-defensin, and alpha-defensin-4 were decreased by H-IPSE exposure. Lipocalin expression is associated with urinary tract infection [38]. Transfection of the bladder urothelium with beta-defensin results in less mucosal damage, edema, and inflammation following UTI induction [39]. These observations may appear puzzling when considered in the context of our findings; H-IPSE-mediated decreases in beta-defensin would thus be thought to enhance bladder damage. However, the functions of anti-microbial peptides are highly redundant, as a number of single anti-microbial peptide gene knockout mice, including beta-defensin-1 [40], have not demonstrated impressive UTI-associated phenotypes [41]. Moreover, inhibition of certain anti-microbial peptides in the setting of UTI has been associated with less bladder damage [42]. Thus, the effects of anti-microbial peptides on the bladder are complex. Finally, to our knowledge, alpha-defensin-4 does not have a critical role in UTI-associated immune responses.

Besides effects on anti-microbial peptide gene expression, IPSE has influence over basophil and mast cell immune responses. This molecule binds to Fcε receptor-associated IgE on the surface of basophils and mast cells [21–23]. Ligation of these complexes could trigger histamine release and anaphylaxis, which are thought to have evolved as part of immune responses to parasites and their products (reviewed by Huang et al. [43]). Understanding IPSE’s ability to trigger histamine release is important to both comprehension of the pathobiology of schistosomiasis and defining this molecule’s safety profile as a potential therapeutic. When H-IPSE was intravenously administered to mice, systemic histamine levels were not elevated at either 5 or 30 min post injection. The 5-min time point is particularly noteworthy considering that histamine release through anaphylactic degranulation is a very rapid physiologic process.

We also sought to characterize systemic aspects of host exposure to H-IPSE besides histamine release. In a repeated daily dose titration of intravenous H-IPSE at up to 2 mg/kg/day, we did not note any obvious adverse effects in terms of body weight trends, feed consumption, hematology, clinical chemistry, or gross pathology. There were very mild alterations in globulin levels and female liver weights (1 mg/kg/day of H-IPSE) that were not clearly significant. We observed sporadic, minimal changes in the kidneys, injection sites, and liver in some animals, but many of these changes were also seen in control animals.

Our findings have noteworthy caveats. For instance, the dosing of H-IPSE may not have been physiologic. Although H-IPSE is one of the most abundant egg-secreted proteins, it is difficult to estimate the exact tissue concentrations of H-IPSE that a *S. haematobium*-infected host would feature. Histamine responses, as well as sensitivity to histamine, are different in mice versus humans. Namely, humans are much more sensitive to histamine than mice. Furthermore, murine basophils have a tenfold lower histamine content (0.1 pg vs 1 pg/cell) compared with the human counterparts [44]. Although we attempted to perform a thorough assessment of any potential systemic pathology caused by H-IPSE exposure, it is possible that an important endpoint was not measured. In addition, differential binding affinities of H-IPSE for IgG and IgE could explain why there is no systemic anaphylactic degranulation. H-IPSE may bind to IgE with higher affinity than to IgG, but given there is several thousand-fold excess of IgG in blood, H-IPSE may be “saturated” with IgG and cannot engage IgE on basophils.

## Conclusions

In conclusion, H-IPSE is not sufficient to induce increased susceptibility to bacterial urinary tract co-infection. This suggests that H-IPSE is either not involved in potential enhanced vulnerability of patients with urogenital schistosomiasis to bacterial urinary tract co-infection or that it works in concert with additional immunomodulatory factors produced by *S. haematobium.* H-IPSE’s apparent lack of toxicity and ability to alleviate UTI-triggered bladder inflammation and edema indicates that it may be suitable for continued development as a potential therapeutic for bladder inflammatory disorders.

## Supplementary information

**Additional file 1.** Additional Tables S1–S21.

## Data Availability

The datasets used and/or analyzed during the current study are available from the corresponding author on reasonable request.

## Abbreviations

A:G: Albumin globulin ratio
C_T_: Cycle thresholds
IgE: Immunoglobulin E
IL-4: Interleukin-4
IPSE: Interleukin-4-inducing principle from *Schistosoma mansoni* eggs
LB: Luria-Bertani
NKT: Natural killer T
NLS: Nuclear localization sequence
PBS: Phosphate-buffered saline
PCR: Polymerase chain reaction
smCKBP: *S. mansoni* Chemokine-binding protein
SD: Standard deviation
UTI: Urinary tract (co-)infections
UPEC: Uropathogenic *Escherichia coli*
