# Supplementary material for: IPSE, a urogenital parasite-derived immunomodulatory molecule, suppresses bladder pathogenesis and anti-microbial peptide gene expression in bacterial urinary tract infection
Source: Parasit Vectors. 2020 Dec 9;13:615. doi: 10.1186/s13071-020-04490-8 (PMC7724859; doi:10.1186/s13071-020-04490-8)
Supplement: Supplementary file 1 — Additional file 1. Additional Tables S1–S21. [file 13071_2020_4490_MOESM1_ESM.docx]

**Table S1: Primer Sequences**

| Gene Name | Sequence |
| --- | --- |
| Uromod | GAC CTG GAT GCT GCT GGT AAT G |
|  | CAC AGC TGC TGT TGG AGC AG |
| TLR5 | GAT CTG CCT CAG AGC ACC TAC G |
|  | GCT ATC CTG CCG TCT GAA GAA CAG |
| TLR4 | GAC ACC AGG AAG CTT GAA TCC CTG |
|  | GAG AGG TGG TGT AAG CCA TGC C |
| LL-37(Camp) | GTG GCT GTG GCG GTC ACT ATC |
|  | CTG CCT TGC CAC ATA CAG TCT CC |
| Lipocalin | CGG CTA GAG ACA AGA GGA AGC TG |
|  | CCG TAC GTG TAG TCC GTG GAC |
| IL-17a | GAT GCT GTT GCT GCT GCT GAG |
|  | CGG TTG AGG TAG TCT GAG GGC |
| IL-12b | GCT GGT GTC TCC ACT CAT GGC |
|  | GCA GGT GTA CTG GCC AGC AT |
| IL-12a | GTT TGG CCA GGG TCA TTC CAG |
|  | CTC TAG TAG CCA GGC AAC TCT CG |
| IL-10 | GCA CTG CTA TGC TGC CTG CT |
|  | CCT GCA TTA AGG AGT CGG TTA GCA G |
| IL-4 | GTA CCA GGA GCC ATA TCC ACG G |
|  | AGA GTC TCT GCA GCT CCA TGA G |
| IFNa | GGT GAT AAG CTA CTG GTC AAC CTG C |
|  | CTG GGT CAG CTC ACT CAG GAC |
| G-CSF | GCA CTA TGG TCA GGA CGA GAG G |
|  | CAG AGA GTG GCC CAG CAA CAC |
| eNOS-3 | CTC CAG CAC CGG AGC CTA G |
|  | GCA CTG AGG GTG TCG TAG GTG |
| c-type lect | CGA AGC GCA CTT CAC AGT GG |
|  | TCT GGG CAT CAC TCT TCA CAT CC |
| b-defensin5 | GCA TTT CTC CTG GTG CTG CTG |
|  | TGC AGC AGT TGA GAC TAG TAA CTC C |
| a-defensin5 | GTC CTC CTC TCT GCC CTT GTC C |
|  | GCA TAT TCA GCT GCA GCA GAA TAC G |
| a-defensin4 | GAC ACT TGT CCT CCT CTC TGC C |
|  | AGT CCC ACG AAC TCG TTC TCC |
| a-defensin2 | CAG GTC CAG GCT GAT CCT ATC C |
|  | AGT CGT CCT GAG CAG GTC CC |
| a-defensin1 | CTG CTT GGC TTC CAG GTC CAG |
|  | CTT TCT GCA GGT TCC ATT CAT GCG |

# Table S2: Summary - Food Consumption (g/Animal) - Male

| **Average Feed Intake/ Animal/ n** | **Treatment Day** | |
| --- | --- | --- |
|  | **1 to 4** | **4 to 7** |
| **Group: G1 Dose: 0 mg/kg/day** | | |
| **Average Feed Intake/ Animal** | 11.2 | 10.2 |
| **n** | 2 | 2 |
| **Group: G2 Dose: 0.5 mg/kg/day** | | |
| **Average Feed Intake/ Animal** | 10.4 | 10.3 |
| **n** | 2 | 2 |
| **Group: G3 Dose: 1 mg/kg/day** | | |
| **Average Feed Intake/ Animal** | 10.5 | 10.9 |
| **n** | 2 | 2 |
| **Group: G4 Dose: 2 mg/kg/day** | | |
| **Average Feed Intake/ Animal** | 10.4 | 9.8 |
| **n** | 2 | 2 |

**Key**: n = Number of Cages.

# Table S3: Summary - Food Consumption (g/Animal) - Female

| **Average Feed Intake/ Animal/ n** | **Treatment Day** | |
| --- | --- | --- |
|  | **1 to 4** | **4 to 7** |
| **Group: G1 Dose: 0 mg/kg/day** | | |
| **Average Feed Intake/ Animal** | 9.0 | 9.5 |
| **n** | 2 | 2 |
| **Group: G2 Dose: 0.5 mg/kg/day** | | |
| **Average Feed Intake/ Animal** | 8.7 | 9.9 |
| **n** | 2 | 2 |
| **Group: G3 Dose: 1 mg/kg/day** | | |
| **Average Feed Intake/ Animal** | 9.2 | 10.0 |
| **n** | 2 | 2 |
| **Group: G4 Dose: 2 mg/kg/day** | | |
| **Average Feed Intake/ Animal** | 8.8 | 9.4 |
| **n** | 2 | 2 |

**Key**: n = Number of Cages.

# Table S4: Summary - Hematology - Male

| **Mean/**  **SD/n** | **WBC** | **RBC** | **HGB** | **HCT** | **MCV** | **MCH** | **MCHC** | **PLT** |
| --- | --- | --- | --- | --- | --- | --- | --- | --- |
|  | **10^3^/µl** | **10^6^/µl** | **g/dL** | **%** | **fL** | **pg** | **g/dL** | **10^3^/µl** |
| **Group: G1 Dose: 0 mg/kg/day** | | | | | | | | |
| **Mean** | 5.17 | 7.73 | 14.07 | 37.53 | 48.53 | 18.20 | 37.43 | 758.33 |
| **SD** | 0.99 | 0.14 | 0.51 | 1.36 | 1.15 | 0.70 | 0.97 | 67.35 |
| **n = 3** |  |  |  |  |  |  |  |  |
| **Group: G2 Dose: 0.5 mg/kg/day** | | | | | | | | |
| **Mean** | 8.17 | 7.56 | 13.97 | 37.10 | 49.07 | 18.50 | 37.70 | 802.00 |
| **SD** | 1.42 | 0.16 | 0.35 | 0.66 | 0.72 | 0.30 | 0.20 | 97.25 |
| **n = 3** |  |  |  |  |  |  |  |  |
| **Group: G3 Dose: 1 mg/kg/day** | | | | | | | | |
| **Mean** | 8.10 | 7.59 | 14.07 | 37.13 | 48.90 | 18.60 | 37.97 | 753.33 |
| **SD** | 2.69 | 0.14 | 0.31 | 0.58 | 0.10 | 0.17 | 0.32 | 47.35 |
| **n = 3** |  |  |  |  |  |  |  |  |
| **Group: G4 Dose: 2 mg/kg/day** | | | | | | | | |
| **Mean** | 6.93 | 7.60 | 14.03 | 37.40 | 49.27 | 18.43 | 37.47 | 734.67 |
| **SD** | 0.59 | 0.32 | 0.51 | 1.45 | 0.15 | 0.15 | 0.25 | 51.60 |
| **n = 3** |  |  |  |  |  |  |  |  |

**Key**: n = Number of animals; WBC = white blood cells; RBC = red blood cells; HGB = hemoglobin; HCT = hematocrit; MCV = mean corpuscular volume; MCH = mean corpuscular hemoglobin; MCHC = mean corpuscular hemoglobin concentration; PLT = platelets

# Table S5: Summary - Hematology - Female

| **Mean/**  **SD/n** | **WBC** | **RBC** | **HGB** | **HCT** | **MCV** | **MCH** | **MCHC** | **PLT** |
| --- | --- | --- | --- | --- | --- | --- | --- | --- |
|  | **10^3^/µl** | **10^6^/µl** | **g/dL** | **%** | **fL** | **pg** | **g/dL** | **10^3^/µl** |
| **Group: G1 Dose: 0 mg/kg/day** | | | | | | | | |
| **Mean** | 5.87 | 7.74 | 14.27 | 37.87 | 49.00 | 18.47 | 37.70 | 698.33 |
| **SD** | 1.08 | 0.15 | 0.12 | 0.64 | 0.66 | 0.29 | 0.35 | 45.08 |
| **n = 3** |  |  |  |  |  |  |  |  |
| **Group: G2 Dose: 0.5 mg/kg/day** | | | | | | | | |
| **Mean** | 7.33 | 7.38 | 13.67 | 36.53 | 49.50 | 18.50 | 37.43 | 711.00 |
| **SD** | 0.42 | 0.23 | 0.68 | 1.44 | 0.92 | 0.46 | 0.40 | 100.64 |
| **n = 3** |  |  |  |  |  |  |  |  |
| **Group: G3 Dose: 1 mg/kg/day** | | | | | | | | |
| **Mean** | 8.47 | 7.12 | 13.37 | 35.50 | 49.83 | 18.80 | 37.70 | 772.33 |
| **SD** | 2.05 | 0.17 | 0.15 | 0.95 | 0.25 | 0.36 | 0.87 | 90.89 |
| **n = 3** |  |  |  |  |  |  |  |  |
| **Group: G4 Dose: 2 mg/kg/day** | | | | | | | | |
| **Mean** | 5.47 | 7.70 | 14.13 | 37.77 | 49.10 | 18.37 | 37.33 | 820.00 |
| **SD** | 0.78 | 0.52 | 0.72 | 1.75 | 1.06 | 0.61 | 0.76 | 22.65 |
| **n = 3** |  |  |  |  |  |  |  |  |

**Key**: n = Number of animals; WBC = white blood cells; RBC = red blood cells; HGB = hemoglobin; HCT = hematocrit; MCV = mean corpuscular volume; MCH = mean corpuscular hemoglobin; MCHC = mean corpuscular hemoglobin concentration; PLT = platelets

# Table S6: Summary - Differential Leukocyte Count (%) - Male

| **Mean/SD/n** | **Neutrophils** | **Lymphocytes** | **Monocytes** | **Eosinophils** | **Basophils** |
| --- | --- | --- | --- | --- | --- |
| **Group: G1 Dose: 0 mg/kg/day** | | | | | |
| **Mean** | 24.33 | 75.00 | 0.67 | 0.00 | 0.00 |
| **SD** | 1.53 | 2.00 | 0.58 | 0.00 | 0.00 |
| **n = 3** |  |  |  |  |  |
| **Group: G2 Dose: 0.5 mg/kg/day** | | | | | |
| **Mean** | 25.00 | 74.67 | 0.33 | 0.00 | 0.00 |
| **SD** | 2.65 | 2.08 | 0.58 | 0.00 | 0.00 |
| **n = 3** |  |  |  |  |  |
| **Group: G3 Dose: 1 mg/kg/day** | | | | | |
| **Mean** | 26.00 | 73.00 | 1.00 | 0.00 | 0.00 |
| **SD** | 2.65 | 3.00 | 1.00 | 0.00 | 0.00 |
| **n = 3** |  |  |  |  |  |
| **Group: G4 Dose: 2 mg/kg/day** | | | | | |
| **Mean** | 24.00 | 75.67 | 0.33 | 0.00 | 0.00 |
| **SD** | 2.65 | 2.52 | 0.58 | 0.00 | 0.00 |
| **n = 3** |  |  |  |  |  |

**Key:** n = Number of animals.

# Table S7: Summary - Differential Leukocyte Count (%) - Female

| **Mean/SD/n** | **Neutrophils** | **Lymphocytes** | **Monocytes** | **Eosinophils** | **Basophils** |
| --- | --- | --- | --- | --- | --- |
| **Group: G1 Dose: 0 mg/kg/day** | | | | | |
| **Mean** | 25.33 | 74.00 | 0.67 | 0.00 | 0.00 |
| **SD** | 3.06 | 2.65 | 0.58 | 0.00 | 0.00 |
| **n=3** |  |  |  |  |  |
| **Group: G2 Dose: 0.5 mg/kg/day** | | | | | |
| **Mean** | 23.67 | 76.00 | 0.33 | 0.00 | 0.00 |
| **SD** | 2.08 | 2.65 | 0.58 | 0.00 | 0.00 |
| **n = 3** |  |  |  |  |  |
| **Group: G3 Dose: 1 mg/kg/day** | | | | | |
| **Mean** | 24.67 | 74.33 | 1.00 | 0.00 | 0.00 |
| **SD** | 2.08 | 2.52 | 1.00 | 0.00 | 0.00 |
| **n = 3** |  |  |  |  |  |
| **Group: G4 Dose: 2 mg/kg/day** | | | | | |
| **Mean** | 25.67 | 73.67 | 0.67 | 0.00 | 0.00 |
| **SD** | 3.51 | 3.06 | 0.58 | 0.00 | 0.00 |
| **n = 3** |  |  |  |  |  |

**Key:** n = Number of animals.

# Table S8: Summary - Reticulocyte Count (%) - Male

| **Mean/SD/n** | **Reticulocyte Count** |
| --- | --- |
| **Group: G1 Dose: 0 mg/kg/day** | |
| **Mean** | 1.80 |
| **SD** | 0.10 |
| **n** | 3 |
| **Group: G2 Dose: 0.5 mg/kg/day** | |
| **Mean** | 1.99 |
| **SD** | 0.10 |
| **n** | 3 |
| **Group: G3 Dose: 1 mg/kg/day** | |
| **Mean** | 2.03 |
| **SD** | 0.21 |
| **n** | 3 |
| **Group: G4 Dose: 2 mg/kg/day** | |
| **Mean** | 1.93 |
| **SD** | 0.21 |
| **n** | 3 |

**Key:** n = Number of animals.

# Table S9: Summary - Reticulocyte Count (%) - Female

| **Mean/SD/n** | **Reticulocyte Count** |
| --- | --- |
| **Group: G1 Dose: 0 mg/kg/day** | |
| **Mean** | 2.09 |
| **SD** | 0.10 |
| **n** | 3 |
| **Group: G2 Dose: 0.5 mg/kg/day** | |
| **Mean** | 1.80 |
| **SD** | 0.10 |
| **n** | 3 |
| **Group: G3 Dose: 1 mg/kg/day** | |
| **Mean** | 1.99 |
| **SD** | 0.30 |
| **n** | 3 |
| **Group: G4 Dose: 2 mg/kg/day** | |
| **Mean** | 1.89 |
| **SD** | 0.10 |
| **n** | 3 |

**Key:** n = Number of animals.

# Table S10: Summary - Clinical Chemistry - Male

| **Animal**  **No.** | **ALT** | **ALB** | **A:G** | **ALP** | **AST** | **BUN** | **CREA** | **CK** |
| --- | --- | --- | --- | --- | --- | --- | --- | --- |
|  | **U/L** | **g/dL** | **-** | **U/L** | **U/L** | **mg/dL** | **mg/dL** | **U/L** |
| **Group: G1 Dose: 0 mg/kg/day** | | | | | | | | |
| **Mean** | 63.57 | 2.90 | 1.60 | 300.87 | 105.03 | 18.78 | 0.15 | 513.60 |
| **SD** | 3.73 | 0.11 | 0.13 | 14.38 | 9.17 | 0.87 | 0.03 | 82.12 |
| **n = 3** |  |  |  |  |  |  |  |  |
| **Group: G2 Dose: 0.5 mg/kg/day** | | | | | | | | |
| **Mean** | 66.17 | 2.83 | 1.60 | 276.20 | 97.60 | 18.60 | 0.16 | 485.63 |
| **SD** | 3.64 | 0.06 | 0.31 | 6.68 | 6.26 | 1.70 | 0.02 | 78.76 |
| **n = 3** |  |  |  |  |  |  |  |  |
| **Group: G3 Dose: 1 mg/kg/day** | | | | | | | | |
| **Mean** | 62.80 | 2.87 | 1.59 | 304.13 | 102.40 | 19.72 | 0.16 | 503.87 |
| **SD** | 8.33 | 0.06 | 0.15 | 22.66 | 5.10 | 1.20 | 0.02 | 57.63 |
| **n = 3** |  |  |  |  |  |  |  |  |
| **Group: G4 Dose: 2 mg/kg/day** | | | | | | | | |
| **Mean** | 63.30 | 2.94 | 1.57 | 295.23 | 100.47 | 19.21 | 0.14 | 532.97 |
| **SD** | 8.49 | 0.15 | 0.11 | 29.15 | 6.17 | 0.94 | 0.01 | 62.89 |
| **n = 3** |  |  |  |  |  |  |  |  |

**Key:** n = Number of animals; ALT = alanine transaminase; ALB = albumin; A:G = albumin to globulin ratio; ALP = alkaline phosphatase; AST = aspartate transaminase; BUN = blood urea nitrogen; CREA = creatinine; CK = creatine kinase

**Table S11: Summary - Clinical Chemistry - Male** Continued…

| **Animal**  **No.** | **GGT** | **GLOB** | **GLU** | **LDLC** | **TBIL** | **TP** | **TGL** | **Urea** |
| --- | --- | --- | --- | --- | --- | --- | --- | --- |
|  | **U/L** | **g/dL** | **mg/dL** | **mg/dL** | **mg/dL** | **g/dL** | **mg/dL** | **mg/dL** |
| **Group: G1 Dose: 0 mg/kg/day** | | | | | | | | |
| **Mean** | 2.25 | 1.83 | 143.10 | 8.45 | 1.73 | 4.79 | 76.47 | 40.20 |
| **SD** | 0.35 | 0.21 | 11.73 | 1.08 | 0.26 | 0.24 | 4.12 | 1.87 |
| **n = 3** |  |  |  |  |  |  |  |  |
| **Group: G2 Dose: 0.5 mg/kg/day** | | | | | | | | |
| **Mean** | 2.11 | 1.82 | 136.93 | 8.34 | 1.57 | 4.66 | 77.13 | 39.80 |
| **SD** | 0.18 | 0.38 | 20.52 | 1.09 | 0.07 | 0.37 | 9.17 | 3.65 |
| **n = 3** |  |  |  |  |  |  |  |  |
| **Group: G3 Dose: 1 mg/kg/day** | | | | | | | | |
| **Mean** | 2.01 | 1.81 | 133.63 | 8.63 | 1.79 | 4.67 | 73.10 | 42.20 |
| **SD** | 0.21 | 0.15 | 20.97 | 1.42 | 0.54 | 0.16 | 14.30 | 2.57 |
| **n = 3** |  |  |  |  |  |  |  |  |
| **Group: G4 Dose: 2 mg/kg/day** | | | | | | | | |
| **Mean** | 2.19 | 1.87 | 138.10 | 9.14 | 1.70 | 4.81 | 82.67 | 41.10 |
| **SD** | 0.14 | 0.06 | 14.53 | 1.51 | 0.36 | 0.15 | 12.53 | 2.01 |
| **n = 3** |  |  |  |  |  |  |  |  |

**Key**: n = Number of animals; GGT = gamma-glutamyl transferase; GLOB = globulin; GLU = glucose; LDLC = low density lipoprotein cholesterol; TBIL = total bilirubin; TP = total protein; TGL = triglycerides

# Table S12: Summary - Clinical Chemistry - Female

| **Animal**  **No.** | **ALT** | **ALB** | **A:G** | **ALP** | **AST** | **BUN** | **CREA** | **CK** |
| --- | --- | --- | --- | --- | --- | --- | --- | --- |
|  | **U/L** | **g/dL** | **-** | **U/L** | **U/L** | **mg/dL** | **mg/dL** | **U/L** |
| **Group: G1 Dose: 0 mg/kg/day** | | | | | | | | |
| **Mean** | 60.97 | 3.01 | 1.86 | 257.53 | 102.13 | 18.85 | 0.16 | 496.60 |
| **SD** | 3.79 | 0.07 | 0.10 | 43.42 | 3.87 | 1.17 | 0.02 | 28.42 |
| **n = 3** |  |  |  |  |  |  |  |  |
| **Group: G2 Dose: 0.5 mg/kg/day** | | | | | | | | |
| **Mean** | 56.27 | 2.89 | 1.60*↓ | 265.67 | 98.10 | 19.66 | 0.16 | 479.83 |
| **SD** | 5.75 | 0.04 | 0.08 | 26.76 | 5.63 | 1.63 | 0.02 | 162.33 |
| **n = 3** |  |  |  |  |  |  |  |  |
| **Group: G3 Dose: 1 mg/kg/day** | | | | | | | | |
| **Mean** | 66.47 | 2.97 | 1.82 | 258.27 | 103.70 | 18.62 | 0.17 | 490.10 |
| **SD** | 5.67 | 0.01 | 0.06 | 50.54 | 5.77 | 1.48 | 0.02 | 31.60 |
| **n = 3** |  |  |  |  |  |  |  |  |
| **Group: G4 Dose: 2 mg/kg/day** | | | | | | | | |
| **Mean** | 59.67 | 2.97 | 1.74 | 266.40 | 103.17 | 19.36 | 0.17 | 512.20 |
| **SD** | 4.06 | 0.08 | 0.05 | 35.52 | 5.20 | 0.99 | 0.01 | 42.65 |
| **n = 3** |  |  |  |  |  |  |  |  |

**Key:** n = Number of animals; ALT = alanine transaminase; ALB = albumin; A:G = albumin to globulin ratio; ALP = alkaline phosphatase; AST = aspartate transaminase; BUN = blood urea nitrogen; CREA = creatinine; CK = creatine kinase; *↓= Mean value of group significantly decreased from control group at p<0.05.

**Table S13: Summary - Clinical Chemistry - Female** Continued…

| **Animal**  **No.** | **GGT** | **GLOB** | **GLU** | **LDLC** | **TBIL** | **TP** | **TGL** | **Urea** |
| --- | --- | --- | --- | --- | --- | --- | --- | --- |
|  | **U/L** | **g/dL** | **mg/dL** | **mg/dL** | **mg/dL** | **g/dL** | **mg/dL** | **mg/dL** |
| **Group: G1 Dose: 0 mg/kg/day** | | | | | | | | |
| **Mean** | 2.17 | 1.62 | 136.10 | 8.56 | 1.61 | 4.63 | 79.03 | 40.33 |
| **SD** | 0.12 | 0.08 | 9.07 | 1.19 | 0.06 | 0.12 | 4.97 | 2.49 |
| **n = 3** |  |  |  |  |  |  |  |  |
| **Group: G2 Dose: 0.5 mg/kg/day** | | | | | | | | |
| **Mean** | 2.09 | 1.80*↑ | 137.93 | 8.37 | 1.67 | 4.69 | 73.03 | 42.07 |
| **SD** | 0.14 | 0.11 | 8.16 | 0.54 | 0.55 | 0.14 | 6.79 | 3.49 |
| **n = 3** |  |  |  |  |  |  |  |  |
| **Group: G3 Dose: 1 mg/kg/day** | | | | | | | | |
| **Mean** | 2.24 | 1.63 | 126.13 | 8.65 | 1.73 | 4.60 | 76.90 | 39.83 |
| **SD** | 0.09 | 0.05 | 9.64 | 1.47 | 0.47 | 0.04 | 5.60 | 3.15 |
| **n = 3** |  |  |  |  |  |  |  |  |
| **Group: G4 Dose: 2 mg/kg/day** | | | | | | | | |
| **Mean** | 2.13 | 1.71 | 132.73 | 8.91 | 1.66 | 4.68 | 80.17 | 41.43 |
| **SD** | 0.24 | 0.01 | 14.17 | 1.01 | 0.14 | 0.07 | 4.63 | 2.10 |
| **n = 3** |  |  |  |  |  |  |  |  |

**Key**: n = Number of animals; GGT = gamma-glutamyl transferase; GLOB = globulin; GLU = glucose; LDLC = low density lipoprotein cholesterol; TBIL = total bilirubin; TP = total protein; TGL = triglycerides; *↑= Mean value of group significantly increased from control group at p<0.05.

# Table S14: Summary - Absolute Organ Weight (g) - Male

| **Mean/**  **SD/**  **n** | **Adrenals** | **Testes** | **Epididymis** | **Liver** | **Spleen** | **Kidneys** | **Heart** | **Thymus** | **Brain** |
| --- | --- | --- | --- | --- | --- | --- | --- | --- | --- |
| **Group: G1 Dose: 0 mg/kg/day** | | | | | | | | | |
| **Mean** | 0.007 | 0.155 | 0.064 | 1.071 | 0.103 | 0.305 | 0.124 | 0.024 | 0.448 |
| **SD** | 0.002 | 0.020 | 0.011 | 0.034 | 0.022 | 0.005 | 0.013 | 0.006 | 0.014 |
| **n = 3** |  |  |  |  |  |  |  |  |  |
| **Group: G2 Dose: 0.5 mg/kg/day** | | | | | | | | | |
| **Mean** | 0.008 | 0.165 | 0.065 | 1.171 | 0.105 | 0.331 | 0.119 | 0.033 | 0.451 |
| **SD** | 0.002 | 0.014 | 0.004 | 0.096 | 0.011 | 0.012 | 0.005 | 0.003 | 0.023 |
| **n = 3** |  |  |  |  |  |  |  |  |  |
| **Group: G3 Dose: 1 mg/kg/day** | | | | | | | | | |
| **Mean** | 0.011 | 0.157 | 0.079 | 1.147 | 0.121 | 0.313 | 0.125 | 0.032 | 0.433 |
| **SD** | 0.002 | 0.013 | 0.028 | 0.147 | 0.012 | 0.027 | 0.017 | 0.007 | 0.026 |
| **n = 3** |  |  |  |  |  |  |  |  |  |
| **Group: G4 Dose: 2 mg/kg/day** | | | | | | | | | |
| **Mean** | 0.011 | 0.166 | 0.057 | 1.170 | 0.110 | 0.313 | 0.122 | 0.028 | 0.438 |
| **SD** | 0.002 | 0.008 | 0.030 | 0.040 | 0.012 | 0.023 | 0.003 | 0.010 | 0.031 |
| **n = 3** |  |  |  |  |  |  |  |  |  |

**Key**: n = Number of animals.

# Table S15: Summary - Absolute Organ Weight (g) - Female

| **Mean/**  **SD/**  **n** | **Adrenals** | **Ovaries** | **Uterus** | **Liver** | **Spleen** | **Kidneys** | **Heart** | **Thymus** | **Brain** |
| --- | --- | --- | --- | --- | --- | --- | --- | --- | --- |
| **Group: G1 Dose: 0 mg/kg/day** | | | | | | | | | |
| **Mean** | 0.009 | 0.017 | 0.051 | 0.846 | 0.103 | 0.220 | 0.099 | 0.041 | 0.439 |
| **SD** | 0.001 | 0.004 | 0.007 | 0.021 | 0.008 | 0.011 | 0.003 | 0.005 | 0.008 |
| **n = 3** |  |  |  |  |  |  |  |  |  |
| **Group: G2 Dose: 0.5 mg/kg/day** | | | | | | | | | |
| **Mean** | 0.010 | 0.018 | 0.097 | 0.834 | 0.102 | 0.209 | 0.096 | 0.035 | 0.422 |
| **SD** | 0.003 | 0.003 | 0.029 | 0.018 | 0.006 | 0.006 | 0.007 | 0.008 | 0.010 |
| **n = 3** |  |  |  |  |  |  |  |  |  |
| **Group: G3 Dose: 1 mg/kg/day** | | | | | | | | | |
| **Mean** | 0.010 | 0.019 | 0.086 | 0.955 | 0.113 | 0.221 | 0.102 | 0.043 | 0.443 |
| **SD** | 0.002 | 0.002 | 0.029 | 0.104 | 0.026 | 0.008 | 0.006 | 0.008 | 0.035 |
| **n = 3** |  |  |  |  |  |  |  |  |  |
| **Group: G4 Dose: 2 mg/kg/day** | | | | | | | | | |
| **Mean** | 0.011 | 0.014 | 0.046 | 0.880 | 0.093 | 0.216 | 0.099 | 0.041 | 0.444 |
| **SD** | 0.002 | 0.002 | 0.012 | 0.122 | 0.013 | 0.019 | 0.004 | 0.004 | 0.024 |
| **n = 3** |  |  |  |  |  |  |  |  |  |

**Key**: n = Number of animals.

# Table S16: Summary - Organ Weight Relative to Body Weight (%) - Male

| **Mean/**  **SD/**  **n** | **Adrenals** | **Testes** | **Epididymis** | **Liver** | **Spleen** | **Kidneys** | **Heart** | **Thymus** | **Brain** |
| --- | --- | --- | --- | --- | --- | --- | --- | --- | --- |
| **Group: G1 Dose: 0 mg/kg/day** | | | | | | | | | |
| **Mean** | 0.034 | 0.756 | 0.313 | 5.242 | 0.504 | 1.494 | 0.605 | 0.116 | 2.192 |
| **SD** | 0.008 | 0.087 | 0.051 | 0.065 | 0.116 | 0.021 | 0.052 | 0.026 | 0.049 |
| **n = 3** |  |  |  |  |  |  |  |  |  |
| **Group: G2 Dose: 0.5 mg/kg/day** | | | | | | | | | |
| **Mean** | 0.037 | 0.801 | 0.311 | 5.630 | 0.505 | 1.594 | 0.577 | 0.159 | 2.170 |
| **SD** | 0.008 | 0.130 | 0.012 | 0.117 | 0.047 | 0.098 | 0.066 | 0.011 | 0.071 |
| **n = 3** |  |  |  |  |  |  |  |  |  |
| **Group: G3 Dose: 1 mg/kg/day** | | | | | | | | | |
| **Mean** | 0.054 | 0.766 | 0.385 | 5.579 | 0.589 | 1.523 | 0.606 | 0.154 | 2.111 |
| **SD** | 0.008 | 0.046 | 0.130 | 0.618 | 0.044 | 0.103 | 0.070 | 0.032 | 0.162 |
| **n = 3** |  |  |  |  |  |  |  |  |  |
| **Group: G4 Dose: 2 mg/kg/day** | | | | | | | | | |
| **Mean** | 0.053 | 0.826 | 0.280 | 5.819 | 0.550 | 1.555 | 0.607 | 0.141 | 2.180 |
| **SD** | 0.011 | 0.033 | 0.145 | 0.158 | 0.071 | 0.087 | 0.007 | 0.048 | 0.175 |
| **n = 3** |  |  |  |  |  |  |  |  |  |

**Key**: n = Number of animals.

# Table S17: Summary - Organ Weight Relative to Body Weight (%) - Female

| **Mean/**  **SD/**  **n** | **Adrenals** | **Ovaries** | **Uterus** | **Liver** | **Spleen** | **Kidneys** | **Heart** | **Thymus** | **Brain** |
| --- | --- | --- | --- | --- | --- | --- | --- | --- | --- |
| **Group: G1 Dose: 0 mg/kg/day** | | | | | | | | | |
| **Mean** | 0.054 | 0.096 | 0.294 | 4.882 | 0.592 | 1.271 | 0.571 | 0.234 | 2.535 |
| **SD** | 0.003 | 0.021 | 0.040 | 0.077 | 0.039 | 0.050 | 0.014 | 0.024 | 0.051 |
| **n = 3** |  |  |  |  |  |  |  |  |  |
| **Group: G2 Dose: 0.5 mg/kg/day** | | | | | | | | | |
| **Mean** | 0.056 | 0.103 | 0.563 | 4.861 | 0.592 | 1.220 | 0.560 | 0.202 | 2.461 |
| **SD** | 0.017 | 0.017 | 0.168 | 0.109 | 0.039 | 0.052 | 0.050 | 0.041 | 0.088 |
| **n = 3** |  |  |  |  |  |  |  |  |  |
| **Group: G3 Dose: 1 mg/kg/day** | | | | | | | | | |
| **Mean** | 0.062 | 0.115 | 0.504 | 5.636*↑ | 0.664 | 1.308 | 0.606 | 0.252 | 2.619 |
| **SD** | 0.014 | 0.018 | 0.142 | 0.287 | 0.110 | 0.066 | 0.075 | 0.030 | 0.159 |
| **n = 3** |  |  |  |  |  |  |  |  |  |
| **Group: G4 Dose: 2 mg/kg/day** | | | | | | | | | |
| **Mean** | 0.062 | 0.082 | 0.262 | 4.998 | 0.528 | 1.226 | 0.564 | 0.231 | 2.528 |
| **SD** | 0.010 | 0.011 | 0.070 | 0.544 | 0.058 | 0.074 | 0.014 | 0.018 | 0.062 |
| **n = 3** |  |  |  |  |  |  |  |  |  |

**Key**: n = Number of animals, *↑= Mean value of group significantly increased from control group at p<0.05.

# Table S18: Summary - Gross Pathology Findings - Male

| **Group** | **G1** | **G2** | **G3** | **G4** |
| --- | --- | --- | --- | --- |
| **Dose (mg/kg/day)** | **0** | **0.5** | **1** | **2** |
| **Number of Animals Examined** | 3 | 3 | 3 | 3 |
| **Mode of Death** |  |  |  |  |
| Terminal sacrificed | 3 | 3 | 3 | 3 |
| **External Abnormalities** |  |  |  |  |
| No abnormality detected | 3 | 3 | 3 | 3 |
| **Internal Abnormalities** |  |  |  |  |
| No abnormality detected | 3 | 3 | 3 | 3 |

# Table S19: Summary - Gross Pathology Findings - Female

| **Group** | **G1** | **G2** | **G3** | **G4** |
| --- | --- | --- | --- | --- |
| **Dose (mg/kg/day)** | **0** | **0.5** | **1** | **2** |
| **Number of Animals Examined** | 3 | 3 | 3 | 3 |
| **Mode of Death** |  |  |  |  |
| Terminal sacrificed | 3 | 3 | 3 | 3 |
| **External Abnormalities** |  |  |  |  |
| No abnormality detected | 3 | 3 | 3 | 3 |
| **Internal Abnormalities** |  |  |  |  |
| No abnormality detected | 3 | 3 | 3 | 3 |

# Table S20: Summary - Histopathology Findings - Male

| **Group** | **G1** | **G4** |
| --- | --- | --- |
| **Dose (mg/kg/day)** | **0** | **2** |
| **Number of Animals Examined** | 3 | 3 |
| **Mode of Death** |  |  |
| Terminal sacrificed | 3 | 3 |
| **Kidney** |  |  |
| Basophilic tubules, focal, minimal | 1 | 1 |
| **Site of Injection** |  |  |
| Infiltration, neutrophil and lymphocytes, focal, minimal | 1 | 0 |

**Note**: Organs/tissues with No Abnormality Detected (NAD) are not included in table.

# Table S21: Summary - Histopathology Findings - Female

| **Group** | **G1** | **G4** |
| --- | --- | --- |
| **Dose (mg/kg/day)** | **0** | **2** |
| **Number of Animals Examined** | 3 | 3 |
| **Mode of Death** |  |  |
| Terminal sacrificed | 3 | 3 |
| **Kidneys** |  |  |
| Basophilic tubules, focal, minimal | 1 | 1 |
| **Liver** |  |  |
| Extramedullary hematopoiesis, focal, minimal | 1 | 0 |
| Cystic space, focal, minimal | 0 | 1 |
| **Uterus** |  |  |
| Atrophy, unilateral, minimal | 0 | 2 |
| **Site of Injection** |  |  |
| Infiltration, neutrophil and lymphocytes, focal, minimal | 0 | 1 |

**Note**: Organs/tissues with No Abnormality Detected (NAD) are not included in table.
